# Application of Design of Experiments in the Development of Self-Microemulsifying Drug Delivery Systems

**DOI:** 10.3390/ph16020283

**Published:** 2023-02-13

**Authors:** Chien-Ming Hsieh, Ting-Lun Yang, Athika Darumas Putri, Chin-Tin Chen

**Affiliations:** 1 School of Pharmacy, College of Pharmacy, Taipei Medical University, Taipei 110, Taiwan; 2 Ph.D. Program in Drug Discovery and Development Industry, College of Pharmacy, Taipei Medical University, Taipei 110, Taiwan; 3 Department of Biochemical Science and Technology, National Taiwan University, Taipei 106, Taiwan; 4 Department of Pharmaceutical Chemistry, Semarang College of Pharmaceutical Sciences (STIFAR), Semarang City 50192, Indonesia

**Keywords:** oral delivery, self-microemulsifying drug delivery system (SMEDDS), quality by design (QbD), design of experiments (DoE)

## Abstract

Oral delivery has become the route of choice among all other types of drug administrations. However, typical chronic disease drugs are often poorly water-soluble, have low dissolution rates, and undergo first-pass metabolism, ultimately leading to low bioavailability and lack of efficacy. The lipid-based formulation offers tremendous benefits of using versatile excipients and has great compatibility with all types of dosage forms. Self-microemulsifying drug delivery system (SMEDDS) promotes drug self-emulsification in a combination of oil, surfactant, and co-surfactant, thereby facilitating better drug solubility and absorption. The feasible preparation of SMEDDS creates a promising strategy to improve the drawbacks of lipophilic drugs administered orally. Selecting a decent mixing among these components is, therefore, of importance for successful SMEDDS. Quality by Design (QbD) brings a systematic approach to drug development, and it offers promise to significantly improve the manufacturing quality performance of SMEDDS. Furthermore, it could be benefited efficiently by conducting pre-formulation studies integrated with the statistical design of experiment (DoE). In this review, we highlight the recent findings for the development of microemulsions and SMEDDS by using DoE methods to optimize the formulations for drugs in different excipients with controllable ratios. A brief overview of DoE concepts is discussed, along with its technical benefits in improving SMEDDS formulations.

## 1. Introduction

Numerous compounds are selected as potential drug candidates by employing high-throughput screening tools. However, >75% of the compounds under current development have poor aqueous solubility [1,2]. In addition, due to the difficulty in disintegrating and dissolving in the gastrointestinal tract, the bioavailability of poorly soluble drugs after oral administration is prone to be low. Physical and chemical modifications of poorly water-soluble drugs have been used to increase their solubility and bioavailability, but there are still some limitations [3,4]. For example, salt form and derivatization may alter the physiochemical properties; however, the change of pH in the physiological environment may lead to drug aggregation or precipitation [5]. Size reduction by micronization could be used to increase the bioavailability of poorly soluble drugs; however, the increased electrostatic interaction between particles may result in difficulties for further compounding and packaging [6]. Recently, lipid-based drug delivery systems, including emulsions [7], microemulsions, self-microemulsifying drug delivery systems (SMEDDS) [8], solid lipid nanoparticle (SLN) [9], nanostructured lipid carrier (NLC) [10,11], and liposome [12], have gained increasing attention for the past decade by virtue of improving the oral bioavailability of poor water-soluble or lipophilic compounds.

## 2. Lipid-Based Formulation for Oral Administration

### 2.1. Lipid Formulation Classification System

The concept of the lipid formulation classification system (LFCS) was introduced by Pouton in 2000 [13] and further well-defined in 2006 [14]. The designation of LFCS depends on the amount of oil (triglycerides or mixed glycerides), surfactant (lipophilic or hydrophilic surfactants), and co-solvent phase. Table 1 shows the four types of compositions and properties of LFCS, which could be used to simulate or interpret different lipid formulations in vivo. Briefly, type I formulations have oils requiring further digestion and emulsification by lipase and endogenous surfactant. This system is suitable for drugs with higher solubility in oils, forming coarse dispersions on dilution. To improve the emulsification and solvent capacities, lipophilic surfactants with hydrophilic-hydrophobic balance (HLB) values of less than 12 are included in type II formulations. However, a continuous phase or coarse emulsion might be found once the content of lipophilic surfactants extends beyond the threshold of 25% (*w*/*w*). In type III formulations, co-solvents are included to blend with oil and surfactants to form a self-emulsifying system. The water-soluble components tend to separate from the oil during dispersion and further dissolve in the water [13]. Moreover, the size of type III formulations easily reaches the nanoscale level after self-emulsification; therefore, these delivery systems are commonly referred to as SMEDDS. Type III formulations are classified into type IIIa and type IIIb. In type IIIa formulations, more amounts of lipids are blended with lipophilic surfactants (HLB < 12) and co-solvents to stabilize the emulsion. In contrast, less amount of lipids are mixed with hydrophilic surfactants (HLB > 12) and/or co-solvents in type IIIb formulations. It has been reported that a fine dispersion with a small particle size (<100 nm) could be produced in the formulations when the amounts of hydrophilic surfactants are over 40% (*w*/*w*) or combined with co-solvents [13]. In this regard, type IIIb can achieve greater dispersion rates with small particle sizes compared to type IIIa formulations. However, drug precipitation might appear in the dispersion process due to the lower lipid content. Type IV formulations do not contain any oil and constitute lipophilic and hydrophilic surfactants. These formulations are suitable for a drug that is hydrophobic but not lipophilic [14]. Since surfactant is mixed with co-solvent in type IV formulations, it provides better solvent capacity on dilution than using co-solvent alone.

### 2.2. The Compositions of Lipid-Based Formulations and Their Role in Enhancement of Bioavailability

#### 2.2.1. Triglycerides and Mixed Glycerides Used as Lipid Phase in Lipid-Based Formulations

Triglyceride is an ester in which three molecules of fatty acid are linked to the alcohol glycerol. Since triglyceride can be completely digested and absorbed after oral administration, the safety concerns are minimized for further pharmaceutical development. Common oils used in the preparation of lipid-based formulation for oral administration are shown in Table 2. Current triglycerides approved by the US Food and Drug Administration (FDA) are mainly derived from plants. According to the length of the fatty acid chain, it could be divided into medium-chain triglycerides (MCT) and long-chain triglycerides (LCT). Basically, MCT is the preferred oil phase used for the preparation of lipid formulations [15,18,19] due to their less suspected oxidative damage [20] and greater solvent capacity compared to LCT [21].

#### 2.2.2. Surfactants

Surfactants are included as an emulsifying agent to avoid phase separation, reduce the interfacial tension and protect the droplets from agglomeration [23]. Presently, the choice of surfactants is still limited due to the safety concern for oral administration. Compared to synthetic surfactants, emulsifiers of natural origin, such as lecithin, have priority for use since they are considered to be safer. Nonionic surfactants are widely used due to the advantages of lower toxicity and irritancy to the GI tract [24], a greater degree of mixing compatibility [25], and maintaining the stability of emulsified vesicles over a wide range of pH or electrolyte [22]. The role of surfactants in these systems is to reduce the interfacial tension and provide sufficient interfacial coverage for microemulsifying the entire oil and water phases [26]. As nonionic surfactants are often used in microemulsions, their selection is very critical, considering the undesirable side effects such as allergy, irritation, or potential intoxication. However, limited references discuss the threshold or maximum dose of nonionic surfactants used in clinicals. Table 3 shows the latest FDA-approved nonionic surfactants with recommended threshold values for lipid-based formulation.

#### 2.2.3. Co-Surfactants/Co-Solvents

Like co-surfactants, co-solvents could regulate the partition of surfactant between the aqueous and oil phases, thereby stabilizing microemulsions to exclude unbounded structures such as liquid crystals, gels, or precipitates [28]. Although both co-surfactants and co-solvents can affect the partition of surfactants, the main role of co-solvents is to accelerate the process of emulsification [29], while co-surfactants is to enhance the interface flexibility of the emulsified vesicle [30]. In general, short to medium-chain length alcohols (C2–C12), ethylene glycol, glycerol, propylene glycol, and other above derivations are adequate co-solvents [22]. Among them, ethanol has been used traditionally as a co-solvent in oral solutions, but it may not be suitable for pediatric or other patients who cannot tolerate alcohol.

### 2.3. Macroemulsions, Microemulsions and Nanoemulsions

**Figure 1:**
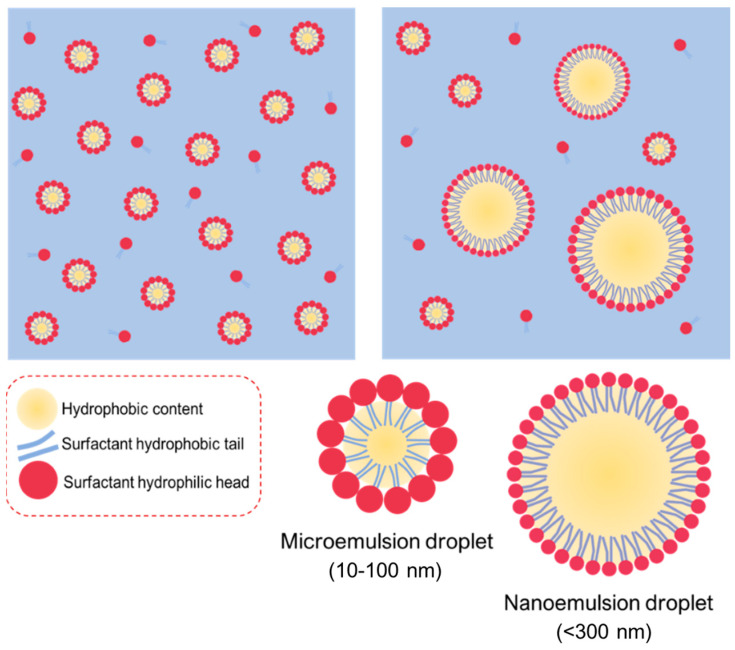
Illustration of microemulsions and nanoemulsions prepared from the similar elements of oil, water, and surfactant, giving the relatively similar structures with each other.

Macroemulsion is a thermodynamically unstable state; therefore, oil-water separation often occurs after storage. If co-solvents such as short-chain alcohols are added during high-speed homogenization, nanoemulsions with particle sizes ranging from 100 nm to 1000 nm can be obtained. When a large amount of surfactant is presented in the oil and water phases, microemulsions with particle sizes ranging from 10 to 100 nm can be formed spontaneously [31,32]. Microemulsions and nanoemulsions are all prepared by oil, water, and surfactant, having relatively similar structures (Figure 1). Owing to the small light scattering of microemulsions and nanoemulsions, the appearance of both is mostly transparent or translucent. However, these two types of emulsions have different composition ratios and formation mechanisms. Microemulsions are formed due to the saturated state of surfactant micelles after a large amount of oil is introduced into them. The free energy of colloidal dispersion is smaller than the separate phase. Therefore, when oil, surfactant, and co-surfactant are blended all together, the microemulsions occur rather spontaneously and involve almost no external energy, further indicating a favorable or stable thermodynamic state. In contrast, the nanoemulsions themselves are usually produced by applying shear stress to induce the formation of nano-sized droplets, resulting in an increase in the interfacial surface free energy. In this regard, nanoemulsions are regarded as thermodynamically unstable because they might further decompose into separate phases over time. However, this mechanism might also offer the nanodroplets to be kinetically stable, which is beneficial for long-term storage. The greater the energy barrier between the initial phase state and the emulsion state, the longer the nanoemulsions last before reverting to their original phase [33].

Particle size is first used to differentiate microemulsions and nanoemulsions. The narrow and sharp peaks refer to microemulsions, whereas the broad or multi-peaks belong to nanoemulsions, suggesting unstable thermodynamics during the nanoemulsion process [33]. Another method for identifying the microemulsions or nanoemulsions is to observe the behavior upon the addition of excess water. In general, a microemulsion is a thermodynamically stable system under a particular range of conditions. However, the system would become unstable during the dilution, and the droplets may break down. Conversely, nanoemulsions are kinetically stable and dilutable with water, which keep the size distribution unchanged with no sign of phase inversion [33].

The advantage of microemulsions and nanoemulsions is their ability to encapsulate lipophilic drugs to enhance their solubility, dissolution (or dispersion) rate, and bioavailability. However, the number of clinical trials related to microemulsions and nanoemulsions decreased over the years [34] due to the large volume and a high proportion of surfactants used in these systems [35]. Table 4 shows the comparison of microemulsions and nanoemulsions.

### 2.4. Self-Microemulsifying Drug Delivery System (SMEDDS)

Emulsion systems are associated with their own set of complexities, including stability and manufacturing problems associated with their commercial production. SMEDDS belongs to lipid-based self-emulsification systems with isotropic appearance. They are promising formulations for delivering poorly water-soluble lipophilic drugs and can spontaneously generate oil-in-water (*w*/*o*) nanosized droplets under gentle blending after dilution in an aqueous medium. Their self-dispersion behavior and small droplet sizes upon dispersion have been shown to improve drug absorption from the large interfacial area. Recently, much attention has been focused on this formulation owing to the ease of manufacture [29], higher drug loading capacity [36,37], and the reduction in food effect [38,39]. Compared to microemulsions and nanoemulsions, SMEDDS can significantly reduce the dose volume, which results in attractive commercial viability and patient compliance. In general, a water-free system not only bears lower solvent effects but also diminishes the dosing volume and increases drug stability. Another advantage of SMEDDS is that drug absorption is less affected by food. Dronedarone is a famous example. Dronedarone is an anti-arrhythmic agent with different bioavailability in fed and fast states [40]. Compared to the fasted state, the AUC_0–24 h_ and C_max_ of the fed state were approximately 10-fold and 8-fold higher, respectively, after oral administration of marketed dronedarone product (Multaq^®^) to beagle dogs [39]. However, SMEDDS formulation significantly mitigated the food effect as AUC_0–24 h_ and C_max_ in the fed state were only 2.9-fold and 2.6-fold higher, respectively. In this regard, it is speculated that SMEDDS may reduce the variability of drug absorption between pre- and post-prandial state, thereby improving therapeutic efficacy and patient compliance.

#### 2.4.1. Formulation Design and Factors Affecting SMEDDS Formulations

SMEDDS formulations consist of mixing aqueous and oily phases in the presence of surfactants and/or co-solvents. Except for the excipient’s selection, several factors are known to influence the formation of a stable SMEDD, such as preparation conditions, equipment conditions, and preparation temperature. Therefore, to develop a successful formulation, it is critical to understand the scientific information behind the system compositions and preparation conditions, which will affect the phase behavior in each excipient.

##### Screening of Excipients

In general, SMEDDS formulations are prepared by mixing different proportions of oil, surfactant, and co-solvent selected by ternary phase diagrams. Construction of ternary phase diagrams is frequently used to determine the types of structures resulting from emulsification and to characterize the behavior of a formulation along a dilution process. After equilibration at atmospheric temperature for a period of time, the drugs are added to the mixture and agitated gently to reach the expected concentration. The appearance of formulations should be transparent and clear without any precipitation. Since external forces are added to accelerate the equilibration during SMEDDS preparation, it is necessary to figure out the sequence of adding the excipients and drugs because it will affect the final appearance. In addition, as the solvent capacity of surfactants in SMEDDS will decrease after solubilizing the drug in co-surfactant [41], the sequence in adding the excipients and drugs not only affect the equilibration of formulation but also the drug solubility.

##### Active Pharmaceutical Ingredient (API) Dose

The SMEDDS is a suitable template for highly hydrophobic APIs, which could dissolve in the oil of formulation. In general, APIs with log *p* larger than five are more suitable to encapsulate in the SMEDDS with high-loading doses [29]. Since SMEDDS belongs to type IIIb lipid-based formulation, more drugs can be loaded into the formulation when higher amounts of surfactant are used. However, if water-soluble constituents are present in SMEDDS, formulation development requires further consideration because it can initiate precipitation of the drug from the formulation into the GI tract medium.

##### Polarity of the Lipid Phase

The digestion of lipid excipients and drug partitioning in SMEDDS begins in the GI tract, involving lipid emulsification and solubilization. During this period, some changes in the properties of the protected APIs in oil droplets could be found [42]. Once the lipase catalyzes the oil droplets, there are differences in the absorption quality and biodistribution of the drug, depending on the lipids sealing it. Then, the drug will be fractionated, dissolved in intestinal fluid, and facilitated by the lipoproteins to transport from the lymphatic system to the blood. Therefore, it is necessary to consider the criterion of lipid selection during SMEDDS formulation.

Caliph and co-workers have compared the triglyceride chains used in SMEDDS. They demonstrated that the combination of medium and long-chain fatty acids improved the droplet formation of microemulsions and increased the bioavailability in 12 h compared to that of using long-chain fatty acid only [43]. Lipids and/or glycerides with longer chains are preferable to act as the oil phase for SMEDDS because they can transform to triglycerides which is more favorable to associate with the chylomicron [44].

#### 2.4.2. Characterization and Evaluation Methods for SMEDDS Formulations

Droplet size is an important parameter in the assessment of SMEDDS since it influences the lipolysis process, drug release, and, consequently, absorption. The droplet size distribution of microemulsion vesicles can be determined by either electron microscopy or light-scattering techniques. The surface charge is determined using a zeta potential analyzer by measuring the zeta potential of the preparations. Zeta potential is the electrical potential in the interfacial double layer of a dispersed particle or droplet versus a point in the continuous phase away from the interface. It is often used as an indicator of droplet stability, where values more positive than +30 mV and more negative than −30 mV indicate good stability against coalescence [45].

The characteristics of SMEDDS not only include droplet size and z-potential but also self-emulsification time, which can generally be evaluated using a USP Type II dissolution apparatus. Briefly, the formulation was added into distilled water maintained at 37 °C, and the time to form a clear solution was recorded with gentle agitation provided at 100 rpm [38]. If the emulsion rapidly forms a clear appearance in less than 1 min, it can be considered as grade I. Grade II indicates the opacity of the emulsion is slightly foggy within 2 min. If a bright white emulsion forms within 3 min, it can be regarded as grade III. Grade IV shows the appearance of dull and grayish-white emulsion with a slightly oily appearance for more than 3 min. In contrast, grade V exhibits poor emulsification with large oil droplets present on the surface [46].

The degree of lipolysis in vitro is also used to evaluate the pre-formulations of SMEDDS. The degradation rate affects the toxicological acceptability and the matrix-controlled release of drugs. In general, lipids digested by lipases to form amphiphilic products are a key process in controlling the utility of most lipid-based formulations. The interaction of these digested products with endogenous amphiphilic components such as bile salts, phospholipids, and cholesterol results in the formation of colloidal structures (e.g., droplet vesicles and micelles). These colloidal structures act as a lipophilic reservoir, enabling the partitioning of drugs into colloidal phases during the gastrointestinal transition. Moreover, exogenous lipids may insert into the bile salts or phospholipid structure, promoting micelle expansion and solubility enhancement. The experimental device consisting of a thermally stable reaction vessel under continuous agitation and a pH-stat with an automated burette to add NaOH solution is used to mimic the in vivo situation of lipolysis. FaSSIF or FeSSIF solution is commonly used as the experimental medium. After lipolysis, the digested mixture is ultra-centrifuged to separate the aqueous phase and sedimentation phase. It is believed that an aqueous phase contains the colloidal structure and dissolved drug, which is imperative for absorption. The sedimentation phase usually contains calcium soap of fatty acid and precipitated drugs, which can be used to evaluate the sedimentation velocity of the lipid-based formulation.

The stability assessment of SMEDDS under different stress can be used to predict their shelf life. As the extra force is included in the SMEDDS manufacturing process, the stability of these formulations depends on the thermodynamic equilibrium. Commonly used experimental tests for stability evaluation include centrifugation tests, freeze-thaw cycle tests, thermal stress tests, and dilution stability [47]. Basically, a SMEDDS pre-formulation is centrifugated for more than 20 min at 3000–13,000 rpm. The appearance of the post-centrifugated suspension was observed and correlated with the size distribution upon self-emulsification in the aqueous. Freeze-thaw cycles are regarded as an experiment to determine the thermal stability of SMEDDS. Some APIs or excipients might be sedimented when SMEDDS is stored at low temperatures. For a stable formulation, the sedimentation should rapidly re-dissolve in SMEDDS as the temperature rises to room temperature. Three freeze-thaw cycles are usually performed on the SMEDDS suspension, including freezing at −20 °C for 48 h and followed by thawing at 40 °C for 48 h. For thermal stress testing, the samples will be placed in a certain temperature range (45 °C to 80 °C) for a period of time to observe whether phase separation occurs. Dilution stability is to evaluate the thermodynamic stability of SMEDDS upon dilution in water. For this purpose, various dilution ratios of the dispersive medium should be tested to determine the consistency of droplet size.

#### 2.4.3. New Strategy for SMEDDS Development

As mentioned above, SMEDDS formulations are used to increase the bioavailability of APIs that are difficult to dissolve and have low bioavailability. Although they are regarded as the most appropriate method to increase drug solubility and bioavailability in oral drug administration, there are still few available products on the pharmaceutical market formulated as SMEDDS. This is associated with the several challenges and difficulties that may be encountered during the SMEDDS preparation and administration process.

API deposition from SMEDDS is one of the most common factors. It is known that drugs encapsulated in SMEDDS must be presented in a dissolved state during transit in the GIT. However, some of the encapsulated drugs are strongly affected by the change of pH values upon contact with GI fluids, resulting in ionization and cancellation of absorption [48,49]. The use of water-soluble solvents or volatile oils may interfere with drug solubility (which increases drug precipitation) when further dilution or high-temperature tests are performed. It is essential for drugs to present in a well-dissolved state in lipid-based delivery. The combined surfactant/co-surfactant and lipid imbalance also increase the possibility of drug precipitation if a greater amount of surfactant/co-surfactant was added than the lipid used in the formulation [50]. The incorporation of polymers to SMEDDS is possible to minimize drug precipitation in vivo [51].

Most of the marketed SMEDDS formulations are in soft gelatin capsules, which causes handling issues and also increases the cost of the product. However, gelatin capsules are associated with few drawbacks. Immature stability can be detrimental from this endeavor as the liquid form is susceptible to possible exposure from hydrolysis, temperature/pH changes, and light, which induce drug/excipient degradation, especially unsaturated lipids as they tend to be oxidized by impurities originating from the gelatin capsule [52,53]. Volatile excipients such as co-solvents in SMEDDS formulations are known to migrate into the shells of soft or hard gelatin capsules, resulting in the precipitation of the lipophilic drugs. Thus, combined polymers and the preparation of solid SMEDDS seems to be a logical solution to address these [54].

The efficiency of oral absorption of the hydrophobic drug from the SMEDDS depends on many formulation-related parameters, such as surfactant concentration, oil/surfactant ratios, the polarity of the emulsion, droplet size, and charge, all of which, in essence, determine the self-emulsification ability. Small changes in material attributes could cause poor product performance in SMEDDS development. The ratio of the oil, surfactant, and co-solvent phases is a key factor in producing a suitable SMEDDS formulation. It has been shown that the formulation efficiency of drugs is affected by the oil/surfactant pairing properties, surfactant concentration, oil/surfactant ratio, and the temperature at which self-emulsification occurs. Therefore, in order to obtain the most efficient self-emulsification zone, the selection of the pharmaceutical excipients is very critical to produce an effective delivery system with better bioavailability. Once a list of suitable excipients is determined, screening of binary drug excipients for solubility, compatibility, and stability will be followed to identify the most appropriate lipid system for the drug in question. According to the LFCS category, SMEDDS can be obtained when the proportions of oil, hydrophilic surfactant, and co-solvent are within <20%, 20–50%, and 20–50%, respectively. However, the range of individual ratios suggested in the LFCS is too wide to find a suitable pre-formulation in a limited time period. Moreover, it can be time-consuming for a formulations scientist to determine the optimal composition of the formulation by a traditional approach.

Quality by design (QbD) is a regulatory-driven approach that adopts a multitude of techniques in product development. This approach can help us to choose the most appropriate component and systemically optimize the formulations. With a controlled and reproducible result, a formulation may meet the specific therapeutic goals. Design of experiment (DoE) and risk assessment techniques based on QbD methodologies are increasingly used in the formulation development of SMEDDS. DoE is a rational and scientific approach for understanding how various formulation/process parameters individually and synergically influence the pivotal product characteristics.

## 3. Overview of the Quality by Design (QbD) and Design of Experiment (DoE) for Pharmaceutical Development

To achieve consistent formulation effects and better quality control, QbD supports parametric options for strong critical attributes. Since reproducibility is a major concern, it is essential to take into account appropriate experimental factors during the variability processing and control or necessarily eliminate a contradictory factor. In other words, it is preferred to ensure the high quality of a product, even though the greater risks are involved, rather than increasing the run quantities [55,56]. Herein, the experiments are not only statistically evaluated (e.g., *t*-test) but also have all the studied parameters analyzed, and the outcomes of those are validated.

### 3.1. Quality by Design (QbD)

QbD in pharmaceuticals involves a systematic methodology incorporated into a series of studies with predetermined objectives, emphasizing the controlled quality of the entire process to produce quality products. Here, risk management is more about how to strategically design and mix inputs and outputs to reduce failure rates. In detail, below are some of the issues in performing pharmaceutical QbD that need to be addressed with reference to the FDA regulations [55,56]: (1) the capability of the selected processes to meet the critical quality attributes, (2) low/minimized product variability amongst the batches, (3) clinical relevance of the developed product specification, (4) efficiency of product manufacture and robustness, and (5) the capability in identifying the problem and management of post-approval change of product.

Several components in QbD include: (1) determining the quality target product profile (QTPP) as critical quality attributes (CQAs) of the developed product, (2) determining the critical material attributes (CMAs) through the design of the product, (3) identifying the critical process parameters (CPPs) through the design of the process and correlating the scale-up principles, CMAs, and CPPs to CQAs, and (4) process capability and continual improvement. By including QbD during the pharmaceutical manufacturing, it is expected that product development could be accelerated with a controlled and measurable risk.

### 3.2. Design of Experiment (DoE)

As mentioned earlier, product and process understanding are key elements of QbD. To best achieve these objectives, in addition to mechanistic models, DoE is an excellent tool that allows pharmaceutical scientists to systematically manipulate factors according to a prespecified design. Traditionally, common experimentation was designed using OFAT (one-factor-at-a-time), which worked by keeping all other variables constant while varying one variable at the same time [57]. Since each experiment must be performed one at a time, numerous runs would be required to achieve adequate information regarding the condition causing the particular problem. Besides being resource (cost, experiments, time, manpower)-intensive, the OFAT method cannot estimate interactions between the variables. DoE, first coined by Ronald A. Fisher in 1935 [58], however, includes all the factors simultaneously by systematic experiments. It has become increasingly prevalent in the formulation arena over the past few years. DoE is a statistical approach to help establish statistical relationships between a set of input and output variables designated by the system/process being studied. Several terms commonly used to describe the flow of DoE include (1) input/independent variables ($x_{1}$, $x_{2}$, $x_{3}$,…), (2) output/dependent variables ($y_{1},y_{2},y_{3}$,…), (3) uncontrollable inputs ($z_{1}$, $z_{2}$, $z_{3}$,…) [59]. Unlike the trial-error method (OFAT), DoE is more efficient and helps structure experiments rationally. The model built by DoE is not only a mathematical model but rather a formal statistical or correlation model that can be derived between input and output variables, wherein each of which is independent.

### 3.3. Screening Experiment and Factorial Design

Many experiments contain various types of parameters/factors with different levels that need to be investigated. Therefore, to make use of DoE involved in the experiments, the possible factors are sorted through the screening experiment, leaving only a few factors having a large effect. The screening stage usually occurs in the early stages of the experiment, where all factors are first considered as likely to have little or no effect on the response. Furthermore, it is important to ensure that the selected factors are presented within their upper and lower limits [57].

To determine the limits, researchers usually use a certain background of the factors studied, for example, based on literature studies or empirical data. The studied factors should then meet factor compatibility, where all the selected factors, any combination amongst, or their upper/lower limits are physically recognized by the system. The determined combinations of the selected factors are expressed as zero points and presented as coordinates in a multi-dimensional factorial space, which is referred to as the zero level [57].

The term ‘interval of factor variation’ refers to the number that will become the upper limit when added to the zero level and will become the lower limit when subtracted from the zero level. In a numerical way, this is usually expressed as +1 as the upper limit (high), −1 as the lower limit (low), and 0 as the central/zero level. These terms later would be used in a factorial design, which is one of the screening methods of DoE to study the effects due to a variable or combination of some factors simultaneously on a response being examined. Geometrically, factorial design collects the data at the vertices of a cube in k-dimensions, wherein **k** is the amount of the studied factors. In full-factorial design (FFD), the data are collected from all the vertices [57,59]. Since this method investigates each factor at 2 levels (i.e., high and low, +1 and −1), therefore it requires 2^k^ experimental runs.

There are circumstances that a particular experiment requires many factors to study. In this case, the fractional factorial design (FrFD) is often used as a strategy against the FFD to deliberately cut the FFD in half [57]. FrFD allows to collect data from specific sub-part of all possible factors, which requires 2^k−q^ runs with –q as the number selected to fractionate the FFD. The most important variable could be identified with this FrFD, allowing for more in-depth tests in the future. FrFD contains several resolutions, and the most important ones are III, IV, and V (regarding the description of each, it has been extensively discussed in another review [60]). FrFD strategy works well in basic designs, such as the most regular fractions, but not in complicated situations, such as some irregular fractions and partial fold designs [59].

In addition, certain fractional factorials have no defining interaction between the factors, such as the Plackett–Burman design (PBD) [59]. PBD is a two-level orthogonal type and is used to develop a proximity fuse [61,62]. The total runs of experiment (N) can be investigated up to N-1 factors with N of multiples of 4 [63,64]. This tool only estimates the main effect of the factors during the process and could not be utilized to obtain surface responses during any optimization [65]. It is recommended to choose a matrix with four or more tests from the selected factors being studied, with three replicates included in the center point of the PBD matrix [64]. Another orthogonal array is the so-called Taguchi method, which is generally similar to fractional factorial experiments. The main objective of this method is to use a standardized method to conduct an experiment and to analyze the results [57,59].

### 3.4. Response Surface Methodology

Response surface methodology (RSM) is a statistical approach used to generate empirical models that typically correlate responses with multiple input factors. It is possible to study the optimization process using the data gathered in this way from the experiments. The y response is a continuous function of several input variables $x_{1}$, $x_{2}$, $x_{3}$,… where the screening design is basically used sequentially to obtain the shape of the response surface [59]. Since the goal of RSM is to find the optimal response, some factors are utilized to obtain the process yield. For instance, in order to find the temperature ($x_{1}$) and pH ${(x}_{2})$ which has an acceptable particle size of SMEDDS (*y*), the approximation can be denoted as follow:

(1) $$y={f(x}_{1},x_{2})+\epsilon$$

ϵ is the noise observed in the response *y*. If the expected response is used herein (i.e., $E(y)={f(x}_{1},x_{2})=\eta$), so the surface will be known as the surface of the response:

(2) $$\eta ={f(x}_{1},x_{2})$$

A screening design performed initially is useful herein to quickly identify which input factors affect the response the most [59]. For example, regarding the maximum drug loading response on SMEDDS parameters such as surface morphology, particle size, and zeta potential can be the most influencing input factors. Analysis of variance (ANOVA) is further used to assess the significance of the combined factors or the influence of their individuals on the response [59].

In RSM, the relationship between response and the factors is apparently not identified yet. Therefore, the steps in doing the RSM begin with finding an appropriate approximation to determine the correct relationship between the response ***y*** and a set of factors *x* [57,59]. Most of the time, it starts with the low-order polynomial. The first-order model is attributed to a well-modeled response with the factors by linear correlation of the factors:

(3) $$y=\beta_{0}+\beta_{1}x_{1}+\beta_{2}x_{2}+\cdots +\beta_{k}x_{k}+\epsilon$$

The second-order model is a polynomial of higher degree which is defined for the system which has curvature:

(4) $$y=\beta_{0}+\sum_{i=1}^{k}\beta_{i}x_{i}+\sum_{i=1}^{k}\beta_{ii}x_{i}^{2}+\sum_{i<j}\sum \beta_{ij}x_{i}x_{j}+\epsilon$$

The $\beta_{0}$, $\beta_{i}$, $\beta_{ii}$, ϵ represent the model constant term, coefficient of the linear term, and coefficient of the quadratic term, respectively. Most of the RSM problems use one or both models to construct the relationship [59,64]. The least squares method is usually then used to estimate the parameters in the polynomial equation. The mathematical model can be considered relevant if the regression is statistically significant and DoEs do not have a meaningful error (lack of fit; usually indicated as *p* > 0.05). Regarding the coefficient of determination (R^2^), it is indicated to be data representative if the coefficient value is closer to 1 [59,64].

### 3.5. Optimization Methodology

**Figure 2:**
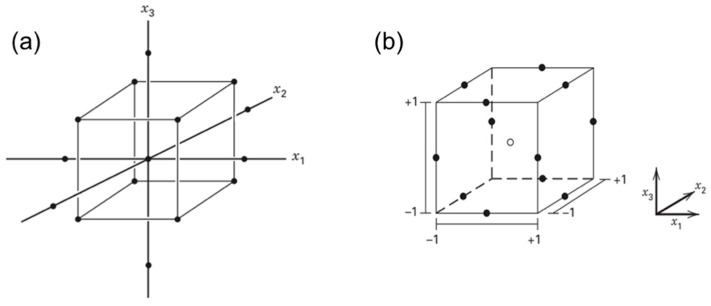
The central composite design (**a**) and Box-Behnken design (**b**) of three factors (*k* = 3). Adapted from Montgomery [59].

RSM is normally employed in the optimization stage of formulation development. Two of the mostly used RSM methods are known as central composite design (CCD) and Box-Behnken design (BBD) (Figure 2). Three-level full factorial design is another type of optimization that is used if two or three input factors are investigated. The number of experiments is set using 3^k^; for example, if there are three input factors, the total runs will be 3^3^, 27 experiments [59]. CCD is one of the examples for fitting a second-order model. It has two level (−1 and +1) factorials with an additional point (axial point or star and center point), which allows for the estimation of the effect of pure squares [59,64]. Mathematically, it consists of 2^k^ factorial with factorial runs of 2^k^ axial or star runs and n_C_ as center runs. The CCD is often used in sequential experimentation, wherein the 2^k^ will be used to fit the first-order model, followed by the axial runs to allow the quadratic terms to be incorporated into the model. Mathematically, it is a selected design to fit the second-order model with the distance α of the axial runs from the design center and the number of center point n_C_. The difference between this design and the factorial design is the presence of a single factor with a coded value in CCD, ±α, varied from 1 to $k^{\frac{1}{2}}$. The α involves rotatability, which depends on the factorial portion of the design. Rotatability is important in RSM. This is because when the optimal location is unknown during optimization, the rotatability acts as the basis for selecting an appropriate design that has the same precision for estimation in all directions [59].

BBD combines 2^k^ factorials with an incomplete block design. It is the three-level design used to fit the response surface. The design is suitable for most of the experiments due to its efficiency and rotatable characteristics. Mathematically, BBD belongs to a spherical design where all the points are on the sphere of radius $2^{\frac{1}{2}}$ [59]. The points are not available at the vertices of the cubic area formed by the upper and lower limits for each variable in the BBD. The number of the experiments is usually counted as $N=2k\left(k-1\right)+Co$ with Co as the number of central points [64].

## 4. Advantages of Applying DoE Techniques for the Development of SMEDDS Formulations

**Figure 3:**
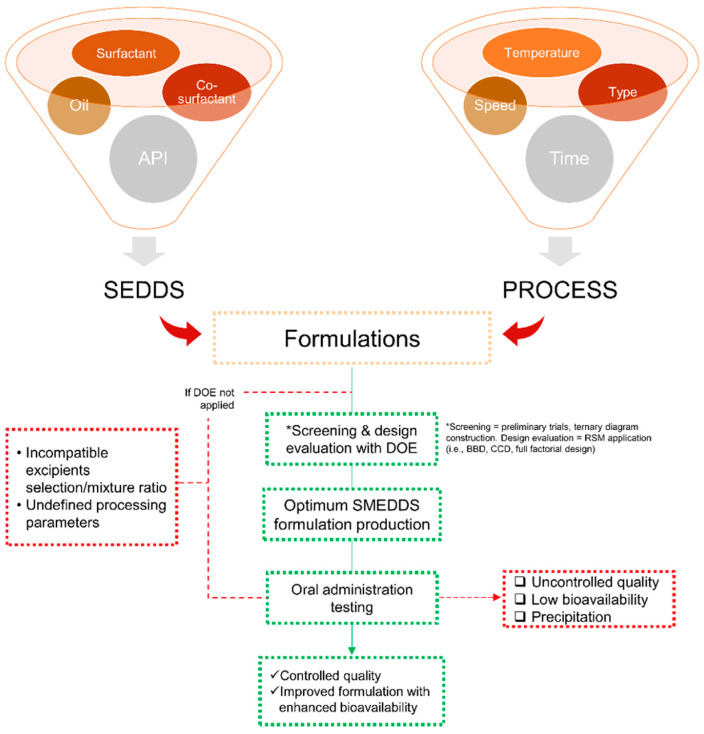
Scheme of the design of experiment (DoE) application in SMEDDS flow-work starting from the selection of materials and processing attributes in a statistical manner to obtain an optimized SMEDDS formulation/parameter. The mentioned abbreviations include self-emulsifying drug delivery (SEDDS), Box-Behnken design (BBD), and central composite design (CCD).

Numerous important parameters need to be involved during the development of SMEDDS formulations, while resources and time are nearly limited. Beyond all that, DoE is one of the effective tools to optimize SMEDDS composition. It offers an efficient experimental formulation that is more rational, ranging from the solubility of the active compound in the combination of excipients, construction of phase diagrams to obtain the most optimal formulation for SMEDDS, all characterizations, and the final responses [66,67,68]. Briefly, an overview of the DoE application in its role in SMEDDS development is presented in Figure 3, wherein it starts from the beginning of the experiments until the evaluation stages.

The following sections are several studies in SMEDDS development using DoE to optimize the variables employed to produce an optimum formulation.

### 4.1. Box-Behnken Design (BBD)

Marasini and coworkers used BBD to investigate the optimum conditions of spray drying parameters for the solid-SMEDDS flurbiprofen formulation [69]. First, the authors conducted a screening study using a spray drying method with dextran as the solid carrier to obtain a range of independent parameter values, including inlet temperature, feed rate, and carrier concentration. Three levels of three-factors (3^3^) BBD were used thereafter to generate a factorial combination of these independent parameters on responses to evaluate powder characteristics, including %moisture, %yield, drug content, and particle size. All parametric factors contributed to influencing the final product characteristics of SMEDDS with a significance value of *p* < 0.05. The most critical factor is the concentration of dextran which has a negative effect on the drug content. The authors showed that the optimized parameter validation of the independent variables was close to the predicted value and could reproduce solid SMEDDS with higher yield (58.5%) and drug content (70.1 mg/g) with minimum moisture content (0.72%) and particle size (166.8 nm).

**Figure 4:**
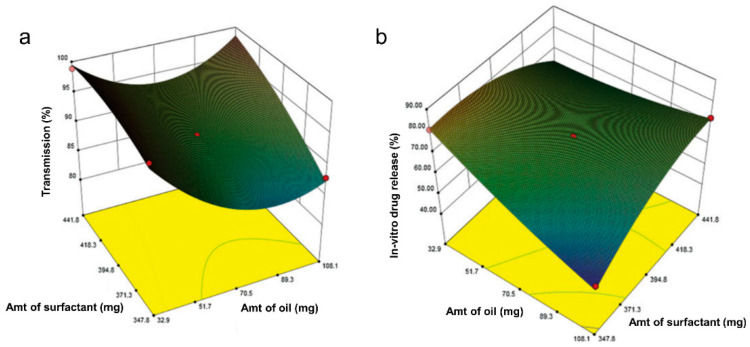
Response surface plot in three dimensions for BBD showing interaction effects between surfactant and oil on % transmission (**a**) and % cumulative drug release (**b**), from the study of zotepine in solid SMEDDS by Dalvadi and coworkers. Adapted from Dalvadi and coworkers [68].

More recently, Dalvadi and coworkers developed zotepine-solid SMEDDS to improve their dissolution rate [68]. Initial screening was performed for the solubility of zotepine in various oil, surfactant, and co-surfactant, which was followed by the construction of pseudo-ternary diagrams to determine the amounts of the selected element. Various solid carriers in different ratios were examined, and Aerosil 200 was chosen as the best one. Three-factor, three-level (3^3^) BBD was then employed to characterize the effect of independent variables (i.e., oleic acid (oil), Tween 80 (surfactant), and PEG400 (co-surfactant)) in the formulation. The % microemulsions transparency and % cumulative drug release were selected based on the principal component analysis as the critical responses used in the BBD. Other variables were also examined, such as the cloud point, emulsification time, and drug content. Irrespective of other variables, the oil content showed an antagonist effect toward both responses significantly, which decreased the % microemulsions transparency and % cumulative drug release (Figure 4a,b). As compared to the conventional zotepine, all optimized parameters produced a higher % transmittance and an improved 30 min-in vitro drug release as final properties of the solid-SMEDDS, which were 98.75% and 86.57%, respectively.

Silva and coworkers developed solid SMEDDS for carvedilol using hot-melt extrusion [70]. Preliminary experiments were done to determine the optimized operating parameters of the extruder with an emphasis on obtaining recirculation time parameters for a homogenous mixing process. BBD was then utilized to evaluate several independent factors, including recirculation time, temperature, and carvedilol concentration, in affecting the cumulative releases in pH 1.2 and pH 6.8. As a result, the increases in recirculation time and temperature significantly lowered the drug release at pH 1.2, while the reduction of both factors increased the release at pH 6.8. In addition, the limited carvedilol solubility significantly affected drug release at pH 6.8, wherein the release was induced if the drug amount decreased. These results underlined another applicability of RSM in constructing efficient and rational variables for system performance used to produce SMEDDS.

Cěrpnjak and coworkers evaluated several solidification methods to produce naproxen solid SMEDDS (tablet), including adsorption, spray-drying, high-shear, and fluid-bed granulation methods [71]. Various carriers were also tested depending on the type of technique to obtain the best solid carrier in transforming the liquid naproxen to a solid state. After obtaining the preliminary results, the spray-drying technique with maltodextrin was selected as the best condition and further used for DoE implementation. The three-factor, two-level (2^3^) factorial design was employed to examine the selected variables, which were inlet temperature, pressure, and pump, on their influences on droplet size, polydispersity index (PDI), and yield. According to the weighted regression coefficients, the antagonistic effects were only indicated in the change of pressure toward the droplet size and the pump speed toward the PDI, whereas the interaction of the three responses had synergistic values. These combined parameters were thus selected to produce the most optimized solid SMEDDS with an inlet temperature of 120 °C, pressure of 50 mmHg, and pump speed of 15 mL/min. Further recent SMEDDS developments governing BBD application are listed in Table 5.

### 4.2. Central Composite Design (CCD)

The central composite design (CCD) is the most commonly used fractional factorial design used in the RSM. It is highly applied in constructing the SMEDDS formulations. The CCD was employed in determining the optimized factors for the osmotic pump capsule developed for SMEDDS [80]. The authors constructed the pseudo-ternary phase diagrams to help examine self-emulsifying regions from various types of oils, surfactants, and co-surfactants, followed by a series of characterizations and analyses. To obtain the optimally controlled release properties, the CCD was done on the elements used in capsule coating, including PEG 400, coating weight, and drug release orifice size. The effect of the independent variables resulted in 81.22% cumulative drug release in 12 with the final formulation of 3% PEG 400, 7.5% coating weight, and 0.5 mm of orifice size. The authors also emphasized the use of lack-of-fit analysis to evaluate critical parameters from the pure error in the replicates (*p* > 0.05).

**Figure 5:**
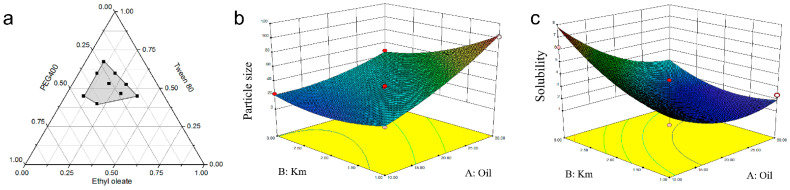
Tertiary-phase diagram showing emulsion areas (in grey color) of the selected masses of the independent variables containing surfactants (i.e., PEG400 and Tween 80 (K_m_)) and oil phases (i.e., ethyl oleate) (**a**). Interaction effects in three-dimensional response surface plots for CCD between K_m_ and oil on particle size (**b**) as well as on solubility (**c**). Adapted from Zheng and coworkers [78].

Zheng and coworkers demonstrated supersaturable-SMEDDS for ellagic acid to improve its solubility [78]. The screening process was done using ternary phase diagram studies which were then followed by the CCD to find the best formulation. Oil phase and surfactant/co-surfactant mixture masses ratio were investigated as the independent factors toward the responses, including particle size and solubility (Figure 5). The decrease of oil mass has an effect on the decrease of particle size, yet reversely for the surfactant mixture (K_m_). In contrast, the oil gives an inverse relationship toward the solubility. Further, the optimized conditions of supersaturable SMEDDS were revealed to be 10% ethyl oleate, 67.5% Tween 80, 22.5% PEG 400, 0.5% PVP K30, and 4 mg/g ellagic acid. The presence of PVP K30 incorporated in the optimized excipients inhibited the precipitation of the drug due to the nucleation effect. The in vitro and in vivo results showed an improved antioxidant ability of ellagic acid in supersaturable SMEDDS formulation.

In addition, Tung and coworkers demonstrated DoE on the selection of excipients to produce pellet SMEDDS containing l-tetrahydropalmatine (l-THP) [81]. The pseudo-ternary diagram was made based on water titration to define the optimum range of Capryol 90, Cremophor RH40, and Transcutol HP as excipients in the selected formulations. The solid carrier employed for pellet SMEDDS was Avicel or Aerosil through extrusion and spheronization techniques. After determining Capryol 90 and the S_mix_ (Cremophor RH40 and Transcutol HP; 3:1) in their best ratio, the central composite face (CCF) design was employed to assess the droplet size, PDI, and dissolution efficiency upon them. As a result, the S_mix_ was indicated to be an antagonist affecting the droplet size and PDI significantly, whereas the Capryol 90 showed a synergistic effect. All responses were well defined according to the optimized parameters with dissolution efficiency of 50%, droplet size of <50 nm, and PDI < 0.3 when using 39.5% capryol 90, 59.2% S_mix_, and 1.3% l-THP to proceed the liquid SMEDDS to the pellet form. Another CCD strategy was used by Yan and coworkers to examine the similar responses (droplet size, PDI, and dissolution efficiency) toward SMEDDS for β-elemene formulation composing poly (acrylic acid) (PAA) entailed on mesoporous silica nanoparticles (MSNPs). The authors emphasized the use of the PAA/MSNPs loaded in SMEDDS to increase the drug loading and to act as the pH triggers in improving a controlled release behavior in an acidic environment [82]. Several reports of CCD applications that have been incorporated in SMEDDS are listed in Table 6.

### 4.3. The Mixture Design

There are other design methods in DoE apart from RSM, which are also widely used in optimizing parameters to be selected in SMEDDS studies, such as the simplex lattice. In contrast to the previous explanation that the levels of the factors are independent, in the simplex lattice, the factors are seen as mixed elements that are not independent. Thus, the simple lattice is categorized as a mixture experiment. Another type of mixture experiment design is a D-optimal mixture, which belongs to the optimality criterion design of the 2k factorial. D-optimal mixture design is available in many commercial software packages and is normally selected if there are design points that need to be further minimized so as to reduce the total time required to produce an optimal design [59]. The applications of these methods also provide key information throughout their results, such as described in Table 7.

Jain and coworkers developed solid SMEDDS for raltegravir potassium, the first line of HIV treatment, by formulating all the selected components within a tablet excipient to improve better stability and dissolution properties of SMEDDS [93]. The simplex lattice method was then employed to rationally design the optimized amounts of independent variables, Labrasol (as oil), Tween-20 (as surfactant), and PEG400 (as co-surfactant). The cumulative percentage of drug release and globular size were examined afterward as the dependent variables. The optimized formulations of SMEDDS were then proceeded with the selected adsorbents to create solid SMEDDS. As a result, the formation of transparent microemulsions of these variables were 50–60% of Labrasol, 20–30% of Tween-20, and 10–30% of PEG400. The presence of either lipid or lipid with co-surfactant interaction greatly affected the cumulative drug release, as shown by the highest coefficient, suggesting that the greater amount of drug was accordingly increased. Meanwhile, lipids with surfactant or lipids with co-surfactant interaction showed a negative coefficient for the globular size, indicating that the increase of either one proportion decreases the globular size of solid SMEDDS to less than 50 nm.

**Figure 6:**
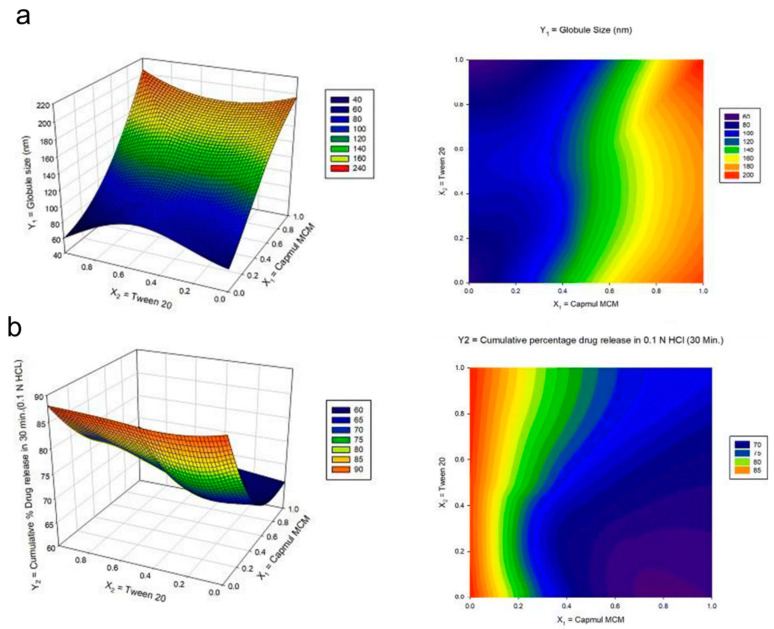
Response surface plot in three-dimension (3D) (left) and the contour plot (right) for the mixture design of the (**a**) globule size and (**b**) cumulative drug release in pH 1.2 media from the study of clofazimine in solid SMEDDS by Dhaval and coworkers. Adapted from Dhaval and coworkers [94].

Another simplex lattice design was also employed by Dhaval and coworkers to investigate seven batches of clofazimine formulations in solid SMEDDS. To do this, the authors used the simplex lattice method to obtain critical parameters toward the responses (particle size and cumulative drug releases in pH 1.2 and pH 6.8) from the screened regions of ternary diagram of the independent variables (Capmul MCM, Tween20, and Labrasol). According to the regression analysis of particle size, the coefficient value of oil was much higher than the other variables, suggesting that the change in Capmul MCM proportion significantly influenced particle size microemulsions. In contrast, the increase of the surfactant (Tween 20) showed a significant decrease of the particle size, as depicted in the 3D response surface and contour plots (Figure 6a). Meanwhile, a lower surfactant level with more oil in pH 1.2 media suggested a decrease in drug release percentage from ~90% to 60% (Figure 6b). A similar trend was found in drug release of pH 6.8 results. Therefore, the authors then concluded to use a high proportion of surfactant to later obtain greater cumulative drug release in the batches studies. The desirability function was then employed to evaluate the closeness of the predicted and actual values obtained from the simplex lattice results. All the designed batches demonstrated acceptable results between the predicted and experimental values (with a bias of <5%), showing the effectiveness of the models. From the optimized parameters, the final cumulative drug release obtained was 85% in less than 60 min at two different dissolution media with a globular size of less than 70 nm.

Lee and coworkers investigated SMEDDS formulations for the BCS IV compound, tolvaptan, through the D-optimal mixture design of DoE [66]. Capryol 90, Tween 20, and Transcutol (or PEG200) were selected for the optimized compositions based on tolvaptan solubility studies. Small particle sizes of <250 nm and an increased cumulative drug release of up to 90% in 60 min were obtained in the formulations involving the oil, surfactant, and co-surfactant with a ratio of 10%, 70%, and 20%, respectively. Their results demonstrate that the successful use of a D-optimal mixture design during the development of tolvaptan-loaded SMEDDS improved the dissolution rate and oral drug bioavailability.

More recently, Na and coworkers carried out SMEDDS to improve the bioavailability of platelet inhibitor, ticagrelor. The authors first performed a preliminary screening to select the optimum excipients from various oils, surfactants, and co-surfactants through solubility and emulsification studies, where the drug in each excipient resulting in greater solubility would be selected. The variables were then selected according to the optimized regions in the pseudo-ternary diagram, including Capmul MCM (oil), Tween 20, or Cremophor EL (surfactant), and Transcutol P (co-surfactant). Scheffe’s mixture design was employed to examine the excipients percentages used with the drug in microemulsions formation toward its solubility, particle size, % transmittance, and % precipitation. As a result, the optimized formulation of ticagrelor in SMEDDS consisting of 10% Capmul MCM, 53.8% Cremophor EL, and 36.2% Transcutol P resulted in maximum values of solubility and % transmittance and minimum values of % precipitation and particle size, along with an exhibited oral bioavailability up to 637.1% as compared to the ticagrelor suspension [95].

There are some reports that only performed the screening process throughout the SMEDDS studies. For instance, Kim and coworkers developed methotreaxate-containing solid SMEDDS [37]. The formulations were done using a spray-drying technique with calcium silicate as the solid carrier. The optimized ratio for castor oil (oil), Tween 80 (surfactant), and Plurol (co-surfactant) were 27:63:10, respectively. The pseudo-ternary diagram was made to assess which formulation could form emulsion simultaneously with a high dissolution rate. As a result, the use of more than 55% surfactant/co-surfactant showed high emulsification efficiency. The methotrexate-containing solid SMEDDS absorption also demonstrated a greater AUC and C_max_ of 2.04 and 3.41 fold, respectively, than the free methotrexate [37].

## 5. Conclusions

SMEDDS have been a popular lipid-based formulation system for the delivery of poorly soluble drugs due to their potential to improve the bioavailability of these active compounds. However, the process of structure formation can be complicated for a complex lipid-based system due to the presence of surfactant, co-surfactant, co-solvent, and carrier that can significantly influence the processing. Using the DoE approach allows formulation scientists to quickly identify interactions between ingredients and reduce the number of experiments required to optimize formulations. As the consequence, scientists have dramatically reduced the time required for formulation development by utilizing this statistical tool. This review illustrates the principles and applications of the most common screening designs applied to SMEDDS development. Furthermore, the use of DoE can be an efficient and fundamental tool to identify and control the variables involved in this scaling-up process to guarantee large-scale production of SMEDDS with the same pharmaceutical activity obtained on the laboratory scale. Finally, the development of SMEDDS by the application of the DoE concept could be a desirable approach to attaining therapeutic and formulary goals.

## Figures and Tables

**Table 1:** The features of four essential lipid formulation types in the lipid formulation classification system [14,15,16,17].

		Type I	Type II	Type IIIa	Type IIIb	Type IV
Composition (*w*/*w* %)	Glycerides (mono-, di-, tri-glycerides)	100	40–80	40–80	<20	0
	Lipophilic surfactants (HLB < 12)	--	20–60	20–40	0	0–20
	Hydrophilic surfactants (HLB > 12)	--	--	0	20–50	20–80
	Co-solvents	--	--	0–40	20–50	0–80
Characteristic features		Oil solution	Self-emulsification	Self-emulsification	Self-micro- emulsification	Spontaneous micelle dispersion
Droplet size		Coarse	0.25–2 µm	100–250 nm	50–100 nm	<50 nm
Lipase digestion		Crucial	Not crucial, but likely	Not crucial, but may occur	Not important	Not important
Disadvantages		Poor solvent capacity for the drugs with log *p* < 2	Coarser emulsion	Possible loss of solvent capacity on dispersion	May cause partial drug precipitation	Risk of drug precipitation upon dispersion

**Table 2:** The characterizations of different types of glycerides used in lipid-based formulations [22].

Class	Example	Characteristics
Medium chain triglycerides (MCT)	Coconut oil Palm seed oil, Miglyol^®^ 812 Captex^®^ 355	Good solubilizing capacity for less lipophilic drugs Higher self-dispersing ability
Long chain triglycerides (LCT)	Corn oil Soybean oil Olive oil Peanut oil Sesame oil Sunflower oil Castor oil	GRAS status Easily ingested, digested, and absorbed Poor self-dispersing properties Lower loading capacity for drugs with intermediate log *p* values Higher solubilizing capacity after dispersion and digestion of the formulation
Mixed mono-, di- and triglycerides	Imwitor^®^ 988 Imwitor^®^ 308 Maisine^®^ 35-1 Peceol^®^ Plurol Oleique^®^ CC49 Capryol^®^ Myrj^®^	Higher self-dispersing ability Higher solubilizing capacity for poorly water-soluble drugs

**Table 3:** Approved nonionic surfactants by the FDA and their descriptions, along with each latest maximum potency per dosage unit per 20 October 2022 [27]. The n/a refers to data not available for the corresponding surfactant.

Surfactants		HLB	Description	Oral	Topical	Injection	Maximum Potency per Dosage Unit
Polyoxylglycerides	Caprylocaproyl polyoxylglycerides (Labrasol^®^)	12	Pale-yellow oily liquids	√	√	--	Oral = 61.2 mg/mL
	Lauroyl polyoxylglyceride (Gelucire 44/14^®^)	11	Pale-yellow waxy solids	√	--	--	Oral = 0.15–218 mg
	Stearoyl polyoxylglycerides (Gelucire 50/13^®^)	11	Pale-yellow waxy solids	√	--	--	Oral = 23.34 mg
Polyoxyethylene Stearates	Polyoxyl 8 stearate	11.1	Waxy cream	√	√	√	Oral = 25 mg/5 mL
	Polyoxyl 12 stearate	13.6	Pasty solid	√	√	√	n/a
	Polyoxyl 20 stearate	14	Waxy solid	√	√	√	n/a
	Polyoxyl 40 stearate	16.9	Waxy solid	√	√	√	Oral = 2–8.48 mg; Topical = 3–8.8% *w*/*w*
	Polyoxyl 50 stearate	17.9	Solid	√	√	√	n/a
	Polyoxyl 100 stearate	18.8	Solid	√	√	√	Topical = 0.5–2.1% *w*/*w*
	Polyoxyl 12 distearate	10.6	Paste	√	√	√	n/a
Polyoxyethylene Sorbitan Fatty Acid Esters	Polyoxyethylene 20 sorbitan monolaurate (Tween 20)	16.7	Yellow oily liquid	√	√	√	Oral = 0.35–4.2 mg; Topical= 0.02–8% *w*/*w*
	Polyoxyethylene 20 sorbitan monopalmitate (Tween 40)	15.6	Yellow oily liquid	√	√	√	Oral = 0.05 mg/5 mL; Topical = 2–3% *w*/*w*
	Polyoxyethylene 20 sorbitan monostearate (Tween 60)	14.9	Yellow oily liquid	√	√	√	Oral = 5–20 mg/mL; Topical = 0.42–14.55% *w*/*w*
	Polyoxyethylene 20 sorbitan tristearate (Tween 65)	10.5	Tan solid	√	√	√	Topical = 0.5% *w*/*w*
	Polyoxyethylene 20 sorbitan monooleate (Tween 80)	15	Yellow oily liquid	√	√	√	Oral = 0.04–418.37 mg; Topical = 0.1–15% *w*/*w*
	Polyoxyethylene 20 sorbitan trioleate (Tween 85)	11	Amber liquid	√	√	√	Oral = 1.5 mg/5 mL
	Polyoxyethylene 20 sorbitan monoisostearate	14.9	Yellow oily liquid	√	√	√	n/a
Polyoxyethylene Alkyl Ethers	Polyoxyl 23 lauryl ether (Brij 35^®^)	16.9	White waxy solid	√	√	--	Topical = 0.45–1.08% *w*/*w*
	Polyoxyl 10 cetyl ether (Brij 56^®^)	12.9	White waxy solid	√	√	--	Topical = 2.5% *w*/*w*
	Polyoxyl 20 cetyl ether (Brij 58^®^)	15.7	Waxy solid	√	√	--	Topical = 2–6% *w*/*w*
	Polyoxyl 10 stearyl ether (Brij 76^®^)	12.4	White waxy solid	√	√	--	n/a
Polyoxyethylene Castor Oil Derivatives	Polyoxyl 35 castoroil (Cremophor EL^®^)	12–14	Pale yellow oily liquid Clear above 26 °C with faint characteristic odor	√	√	√	Oral = 0.4–515 mg/mL Topical = 4% *w*/*w*
	Poloxyl 35 castoroil, purified (Cremophor ELP^®^)	12–14	White to slightly yellowish paste or cloudy liquid with weak characteristic odor	√	√	√	n/a
	Polyoxyl 40 hydrogenated castoroil (Cremophor RH40^®^)	14–16	Viscous liquid or soft paste with very little odor in aqueous solutions, almost tasteless	√	√	√	Oral coated capsule = 101.25 mg Oral solution = 450 mg/mL Topical = 1% *w*/*w*
	Polyoxyl 60 hydrogenated castor oil	15–17	White to yellowish soft or flowing paste with faint odor or taste in aqueous solutions	√	√	√	Topical = 1.9% *w*/*w*
D-α-Tocopherol polyethylene glycol 1000 succinate (TPGS)		13.2	White to light-brown, waxy solid	√	√	--	n/a

**Table 4:** The comparison of nanoemulsions and microemulsions [22,32].

	Nanoemulsions	Microemulsions
Stability	Kinetic stable system	Thermodynamic stable system
Compositions	Oil, Surfactants, Water	Oil, Surfactants, Water
Order of mixing	The surfactant should first be mixed with the oil phase, and then titrated with the aqueous	The order of mixing does not affect the size of particle
Particle size	50–300 nm	10–100 nm
Manufacturing process	Specific equipment is required to provide sufficient energy to increase the interfacial area	Spontaneous formation

**Table 5:** List of the SMEDDS developments along with the applied response surface methodology (RSM) of central composite design (CCD).

Compound	Screening	RSM	Experiments	Independent Variables	Responses	Program	Optimized Conditions	Reference
6-Shogaol (purified alkylphenol from ginger root)	n/a	CCD	*p* < 0.05	Ethyl oleate (18.62% *w*/*w*), tween 80:PEG 400 (1.73:1 *w*/*w*)	Particle size, PDI, cumulative drug release	Design-Expert^®^, version 8.0.6	Particle size (20.00 ± 0.26 nm), PDI (0.18 ± 0.02), increased cumulative release compared to free 6-shogaol, oral bioavailability	[72]
Lornoxicam	Regular experiment	CCD	*p* < 0.05	Labrafil M 1944 CS (25%), Kolliphor HS 15 (56.25%), Transcutol HP (18.75%)	Particle size, PDI, self-emulsifying time	Design-Expert^®^ n/a version	Particle size (70.14 ± 1.06 nm), PDI (0.193 ± 0.010), self-emulsifying time (68 ± 2 s)	[73]
Chrysin	Compatibility tests and pseudo-ternary phase diagram studies	CCD	*p* < 0.05	Surface morphology, pH, diameter, PDI, zeta potential, and phase type	Maximum drug loading and optimize SMEDDS formation	Design-Expert^®^ n/a version	Medium chain triglyceride:oleic acid:Cremophor RH40: Transcutol HP *w*/*w*) (12%:12%:32%:44%), with a drug loading capacity of 5 mg/g	[74]
Phillygenin	Compatibility tests and pseudo-ternary phase diagram studies	CCD	*p* < 0.05	Oil phase mass% and surfactant/co-surfactant mixture weight ratio	Equilibrium solubility, particle size, PDI	Design-Expert^®^ version 8.0.6	Optimized Labrafil M1944CS:Cremophor EL:PEG400 = 27.8:33.6:38.6% wt produced 10.2 mg/g equilibrium solubility, 40.11 ± 0.74 nm particle size, and 0.243 ± 0.01 PDI	[75]
Luteolin	Compatibility tests and pseudo-ternary phase diagram studies	CCD	*p* < 0.05	Weight percent of oil and the mass ratio	Particle size, PDI, self-emulsifying time	Design-Expert^®^ version 8.0	Optimized Crodamol GTCC:Kolliphor EL:PEG400 = 20.1:48.2:31.7% wt produced LUT loading capacity = 24.66 mg/g; S-SNEDDS showed 2.2-fold increase of bioavailability compared to conventional SNEDDS.	[76]
Triptolide	n/a	CCD	n/a	Oil phase mass% and surfactant/co-surfactant mixture weight ratio	Particle size and drug content	Design-Expert^®^ version 8.0.6	Optimized MCT:EL:PEG400 = 25.3:49.6:25.1 with particle size of 30.46 nm and drug content of 2.91 mg/g. These optimized parameters produced SMEDDS with complete release in 6 h, increased oral bioavailability, and enhanced the tumor inhibitory effect.	[77]
Ellagic acid	Ternary phase diagram studies	CCD	*p* < 0.01	Oil phase mass% and surfactant/co-surfactant mixture weight ratio	Particle size and solubility	Design-Expert^®^ version 8.0.5	10% ethyl oleate, 67.5% Tween 80, 22.5% PEG 400, 0.5% PVP K30 and 4 mg/g ellagic acid. The presence of PVP K30 in the optimized excipients inhibited the precipitation. The in vitro and in vivo showed an improved antioxidant ability of eligilic acid.	[78]
Rhubarb free-anthraquinone	n/a	CCD	*p* < 0.05	Mass ratio of Neusilin US2/preconcentrated RhA nanoemulsions and contents of PVPP % *w*/*w*	Friability, disintegration time, and 4 h cumulative dissolution rate of RhA in SNEDDS tablets	Design-Expert^®^ version 8.0.6	Optimized 1:1(*w*/*w*) Neusilin US2/pre-concentrated RhA nanoemulsions, 5.0% *w*/*w* PVPP, 1% *w*/*w* Mg stearate produced friability of 0.389 ± 0.007%, disintegration time of 5.13 ± 0.14 min, and 4 h-dissolution rate of 87.91 ± 1.89%.	[79]

**Table 6:** List of the SMEDDS developments along with the applied response surface methodology (RSM) of Box-Behnken design (BBD).

Compound	Screening	RSM	Experiments	Independent Variables	Responses	Program	Optimized Conditions	Reference
Furbiprofen	Regular experiment	BBD (3^3^)	*p* < 0.05	Inlet temperature, feed rate, and carrier concentration	%moisture, %yield, drug content, and particle size	Design-Expert^®^ version 8.0.5	%yield (58.5%) and drug content (70.1 mg/g) with minimum moisture content (0.72%) and particle size (166.8 nm).	[69]
Zotepine	Pseudo-ternary diagrams studies	BBD (3^3^)	*p* < 0.05	Oleic acid (oil), Tween 80 (surfactant), and PEG400 (co-surfactant)	%microemulsions transparency and %cumulative drug release	Design-Expert^®^ version 8.0.5	%transmittance of 98.75% and an improved 30 min-in vitro drug release of 86.57%.	[68]
Dapsone	Pseudo-ternary diagrams studies	BBD (3^3^)	*p* < 0.05	Inlet temperature, feed flow rate, carrier concentration	Particle size and %yield	Design-Expert^®^ version 11.0	The optimized solid SMEDDS with inlet temperature of 130 °C, flow rate of 6 mL/min, and carrier conc. (i.e., neusilin US2) of 0.25% resulted in 87.5 ± 4.95 nm of particle size and yielded 34.06 ± 1.70%.	[83]
Carvedilol	Regular experiments on formulation compositions and hot melt extruder conditions	BBD (3^3^)	*p* < 0.05	Recirculation time, first heating zone temperature, API concentration	%drug releases (in 0.1 M HCl and 0.4 M phosphate buffer), %efficiency, and particle size	Statistica ^®^ version 7.0	The optimized formulation of carvedilol in solid SMEDDS using hot-melt extrusion resulted in max. 25.54 ± 0.77% release in HCl followed by max. 85.54 ± 1.79% release in phosphate buffer.	[70]
Fenofibrate	Pseudo-ternary diagrams studies	BBD (3^3^)	*p* < 0.05	Amount of Labrafil M 1944 (oil), Labrasol (surfactant), and Capryol (co-surfactant)	Particle size, %cumulative release in 30 min, and equilibrium solubility	Design-Expert^®^ version 8.0.4	The optimized formulation of fenofibrate in solid SMEDDS resulted in 113.13 ± 1.63 mg/g solubility with particle size of 171.4 ± 2.5 nm, %cumulative release of 87.7 ± 1.6%, and 3.6-fold higher bioavailability than its free-form suspension.	[84]
Ezetimibe	Pseudo-ternary diagrams studies	BBD (3^3^)	*p* < 0.05	Amount of Peceol (oil), Tween 80 (surfactant), Transcutol P (co-surfactant)	Particle size, %transmittance, self-emulsification time, %cumulative releases in 5 and 40 min	Design-Expert^®^ version 11.0	The optimized ezetimibe in solid SMEDDS resulted in 26.31 ± 2.64 nm particle size, 69.26 ± 2.56 self-emulsification time, and 95.38 ± 3.67% cumulative release in 40 min.	[85]
Naproxen	Regular experiment	FFD	*p* < 0.05, except %yield	Inlet temperature, pressure, and pump speed	Droplet size, PDI, and %yield	Unscrambler1 software(version 10.1, CAMO software)	The inlet temperature of 120 °C, pressure of 50 mmHg, and pump speed of 15 mL/min resulted the optimized solid SMEDDS.	[71]

**Table 7:** List of the SMEDDS developments along with the applied response surface methodology (RSM) of D-optimal mixture design.

Compound	Screening	RSM	Experiments	Independent Variables	Responses	Program	Optimized Conditions	Reference
HL235 (i.e., Cathepsin K inhibitor)	Pseudo-ternary diagrams studies	D-optimal mixture	*p* < 0.05	Capmul MCM (oil), Tween-20 (surfactant), Carbitol (co-surfactant)	Cumulative drug release in 15 min and solubilization capacity	Design-Expert^®^ version 7.0	The optimized SMEDDs formulation resulting in 2.34 ± 0.21 µg/mL and solubilization capacity of 6.164 ± 0.06 mg/mL.	[86]
Blonanserin	Pseudo-ternary diagrams studies	D-optimal mixture	n/a	Captex 200P: Capmul MCM (1:1) (oil), Tween-20 (surfactant), and ethanol (co-surfactant)	Drug loading, percentage cumulative drug release, particle size	n/a	The optimized Blonanserin in SMEDDS with 1:1 (23% *v*/*v*) Captex 200P:Capmul MCM mixture, Tween-80 (57% *v*/*v*), and ethanol (20% *v*/*v*) produced cumulative drug release of 94.72% in 30 min and particle size of 21 nm	[87]
Olmesartan medoxomil	Pseudo-ternary diagrams studies	D-optimal mixture	*p* < 0.05	Capmul MCM EP (oil), Kolliphore EL (surfactant), Transcutol P (co-surfactant)	Cumulative drug release and particle size	JMP ver.9.0.0 software	The optimized formulation with Capmul MCM EP (23% *v*/*v*), Kolliphore EL (49% *v*/*v*) and Transcutol P (28% *v*/*v*) resulted in 94.7% of drug release and 105 nm of particle size.	[88]
Telmisartan (loaded with phospholipid complex)	Pseudo-ternary diagrams studies	D-optimal mixture	*p* < 0.05	Capryol 90 (oil), Tween 80 (surfactant), and tetraglycol (co-surfactant)	Drug loading, drug release, and particle size	Minitab ver.17.0 software	The optimized SMEDDS formulation of telmisartan loaded phospholipid complex resulted in 22.17 nm of globular size, 4.06 mg/mL of solubilization, and 99.4% of drug release in 15 min.	[89]
Curcumin and artemisin	Pseudo-ternary diagrams studies	D-optimal mixture	*p* < 0.05	Oleic acid (oil), Tween-80 (surfactant), and PEG400 (co-surfactant)	%transmittance, particle size, and polydispersity index	Design-Expert^®^ version 10.0	The optimized SMEDDS containing curcumin and artemisin produced 98.27% of transmittance, 150.7 nm of particle size, and 0.118 of polydispersity index.	[90]
Ziyuglycoside I	Solubility and pseudo-ternary diagrams studies	D-optimal mixture	*p* < 0.05	Obleique CC497 (oil), Tween-20 (surfactant), and Transcutol HP (co-surfactant)	Drug loading and particle size	Design Expert version 8.0.4.1	An enhanced solubility up to 23.93 mg/g and particle size of 207.92 ± 2.13 nm, along with an improved bioavailability (21.94%) as compared to the free drug (3.16%)	[91]
Insulin	Solubility and pseudo-ternary diagrams studies	D-optimal mixture	*p* < 0.05	Capmul MCM (oil), Labrasol (surfactant), Tetraglycol (co-surfactant)	Particle size, stability, and leakage	Design-Expert version 11.0	The optimized insulin in SMEDDS formulation resulted in particle size of 115.2 nm, enhanced stability up to 46.75%, and lessened leakage down to 17.67%	[92]

## Data Availability

Data sharing is not applicable.
